# LCIA Formatter

**DOI:** 10.21105/joss.03392

**Published:** 2021-10-10

**Authors:** Ben Young, Michael Srocka, Wesley Ingwersen, Ben Morelli, Sarah Cashman, Andrew Henderson

**Affiliations:** 1 Eastern Research Group, Inc.; 2 GreenDelta; 3 U.S. Environmental Protection Agency

## Abstract

Life Cycle Assessment (LCA) is an established and standardized methodology to comprehensively assess environmental and public health metrics across industries and products (International Organization for Standardization, 2006). The United States Environmental Protection Agency (USEPA) is developing an open source LCA tool ecosystem (Ingwersen, 2019). The ecosystem includes tools to automate the creation of life cycle inventory (LCI) datasets, which account for flows to and from nature for steps across the life cycle of products or services, and tools for life cycle impact assessment (LCIA) to support classification and characterization of the cumulative LCI to potential impacts. Impacts are expressed via indicators, either midpoint or endpoint, corresponding to different points on the environmental cause-effect chain model (Frischknecht & Jolliet, 2016). This paper describes a USEPA LCA ecosystem tool ‘LCIA formatter’ that extracts LCIA information from original source methods and converts the data for interoperability with the rest of the USEPA LCA ecosystem tools.

## Statement of need

A simplified algorithm for LCA is given in Equation 1, where *I* are impacts, *E* are emissions generated (e.g. pollutants) or raw resources consumed (e.g. land, water) per functional unit of product across the entire supporting product system and *CF* are corresponding characterization factors that quantitatively relate a unit of a flow to a given impact indicator (e.g., global warming, acidification, total land use, etc.).

(1) I=∑(E*CF)

Both *E* and *CF* use objects called elementary flows, which are data objects that generally represent a substance (e.g. Ammonia), source or receiving environmental context (e.g. Freshwater lake), and unit (e.g. kilogram). *E* will have a total quantity of a given elementary flow from the product system, and the characterization factors in *CF* are in the form of the indicator unit per elementary flow unit (e.g. kg N-eq per kg Ammonia). *E* comes from the LCI calculated for the given product under user-defined conditions, and *CF* is a static dataset that comes from an LCIA method provider.

LCA software generally include LCIA methods to provide impact assessment results for user-created and software-provided LCI, but the elementary flows used in these LCIA methods must be the same data objects as the flows in the LCI data to enable proper impact result calculations. A major challenge to reproducing LCA results and sharing LCA models across platforms is that there is no internationally common list of elementary flows used by either LCI, LCIA, or software providers. A critical review of elementary flow data used in LCA showed that flows in LCIA methods are the least clearly described and therefore least portable and machine-readable among those from LCI, LCIA and software providers (Edelen et al., 2018). Furthermore, LCA software providers of both proprietary and open source software rarely provide an open process or external peer review of their incorporation of data from LCIA providers (Brightway2 is one exception (Mutel, 2017)). This is one potential cause of discrepancies in results across LCA software.

The LCIA formatter is a specific solution to this problem. The LCIA formatter transparently acquires LCIA methods from original provider data portals, maps them to an authoritative flow list, and exports them in common data formats. The LCIA formatter v1.0 uses the Federal LCA Commons Elementary Flow List (FEDEFL) as the authoritative system of elementary flows (Edelen et al., 2019). This system has been adopted by federal agencies in the U.S. to share data through the Federal LCA Commons (FLCAC). The automation of this process provided by the LCIA formatter is critical, because as elementary flows used in LCI are updated, the LCIA datasets should also be automatically updated, and vice versa, to facilitate their use in LCA.

## Structure

The LCIA formatter code is written in Python 3 and created as a standard python package called lciafmt that can be installed using pip. The LCIA formatter primarily uses pandas (McKinney, 2010) for data wrangling, the Apache parquet format (Apache, 2021) for local storage of processed datasets, and olca-ipc (Srocka, 2020) for writing data in a standard LCA data format. The code is stored on a USEPA GitHub repository and is publicly accessible.

The LCIA formatter accesses LCIA datasets directly from the data provider. These datasets are typically provided as Microsoft Excel or Access files. These are downloaded and saved in a temporary local cache. To support the specific functions necessary to access and parse individual methods, each method is processed within its own Python module. Flow names, indicators, characterization factors, and other metadata are compiled in a standard format. Adjustments are made as needed to improve consistency between indicators and across methods. This includes handling duplicate entries for the same elementary flow and data cleaning (such as cleaning string names, adjusting capitalization, formatting of CAS Registry Numbers). Additionally, the LCIA formatter supports the inclusion of non-specified secondary contexts (emission locations) where none are provided. Where methods provide both midpoint and endpoint categories within a single source, the LCIA formatter parses these indicators for separate use. Midpoint indicators are typically calculated at an intermediate point along the cause-effect model (such as final mass of substance in the environment), and endpoint indicators typically correspond to damage from the elementary flows (such as a disability-adjusted life year, or DALY) (Frischknecht & Jolliet, 2016). Therefore, midpoint and endpoint indicators should be evaluated separately. Finally, source flow data are mapped to elementary flows in the FEDEFL (Edelen et al., 2019), through mapping files provided within that package (Ingwersen et al., 2020). These mapping files correspond to flow names and contexts to a common set of elementary flows generated for LCA modeling by the USEPA. Because flows are provided at more detailed contexts than exists in most LCIA methods currently, mapping to the FEDEFL typically expands the number of characterization factors available. Mapped methods are stored locally as parquet files for future access by LCIA formatter or other tools. Additionally, mapped methods can be exported in JSON-LD format for use in LCA software tools such as openLCA.

## Available Methods

The LCIA formatter is structured to easily convert original source data from existing LCIA methods. Version 1.0 of the LCIA formatter converts three commonly used LCIA methods: TRACI 2.1 (Bare, 2011), ReCiPe2016 (Huijbregts et al., 2017), and ImpactWorld+ (Bulle et al., 2019) (Table 1), which cover a variety of impact categories. Cumulative LCI indicators based on the FEDEFL are also available as an output of the LCIA formatter.

### TRACI2.1

USEPA’s Tool for Reduction and Assessment of Chemicals and Other Impacts (TRACI) is widely used for LCA across the U.S. Federal Government and by U.S.-based LCA practitioners (Bare, 2011). The LCIA formatter accesses the TRACI Excel-based source file and characterizes impacts across 9 midpoint indicators for use in U.S.-focused analyses. When available, flow characterization factors are mapped to the release context with the greatest level of detail. For example, an air emission may be linked to the specific release height and population density of release area. If secondary context data is unknown, the LCIA formatter generates an average factor across the possible contexts to ensure the flow will still be captured in model calculations.

### ReCiPe2016

ReCiPe 2016 characterizes impacts across 18 midpoint indicators and three perspectives: Individualist, Hierarchist, and Egalitarian (Huijbregts et al., 2017). The LCIA formatter generates endpoint impacts through a series of midpoint conversion factors provided for each indicator. As is done for TRACI, where characterization factors are not supplied for secondary contexts, an average factor across the possible contexts is generated. This ensures that users that do not specify a secondary context (e.g. emission to air with no indication of population density) can still obtain a characterization factor for a flow.

### ImpactWorld+

ImpactWorld+ v1.3 provides characterization factors for 44 indicators at midpoint or endpoint levels (Bulle et al., 2019). ImpactWorld+ v1.3 is downloaded as an Access database and read into a pandas dataframe using pyodbc. Only elementary flows with global characterization factors are included at this time, as spatially explicit characterization factors (e.g., country) are not yet compatible with the FEDEFL. Context information is added for water scarcity and availability categories. Flowable name is applied as context for land occupation and transformation categories. Context descriptions are provided in the original source for all other categories.

### FEDEFL Inventory Methods

The LCIA formatter generates life cycle inventory methods based on groups of elementary flows identified in the FEDEFL. For example, an inventory method for energy resource use represents the summation of all instances of these flows within a dataset. Where necessary unit conversions are applied to achieve a consistent indicator unit.

### Valuation

The LCIA formatter also includes a method-agnostic approach to convert indicators (midpoint or endpoint) to monetary values. The primary valuation is based on modified budget constraint modeling (Weidema, 2009), updated to USD2014. A DALY is valued as the global average annual potential economic production per capita; the value of ecosystem damages is calculated from the ratio of global population to terrestrial surface area, and it is also validated based on environmental preservation expenditures in selected countries. The conversions between the different ecosystem impact indicators (e.g., PDF.m2.yr and species.yr) are based on the species density calculations from ReCiPe 2008 (Goedkoop et al., 2009).

## Applications

The LCIA methods generated by the LCIA formatter for use with the FEDEFL are hosted publicly on the FLCAC for use by LCA practitioners and researchers. These methods support LCAs performed by many parties, including member agencies for the Federal LCA Commons such as USEPA, U.S. Department of Energy, U.S. Department of Agriculture, U.S. Department of Defense, and others. These methods also enable impact assessment for researchers utilizing the US Life Cycle Inventory (USLCI) Database. LCIA methods from the LCIA formatter are also being used in the standard format as inputs into other USEPA LCA ecosystem tools, including useeior (Ingwersen et al., 2021) and the Electricity Life Cycle Inventory (electricitylci) (Jamieson et al., 2020). The system was built to be flexible enough to support creating outputs for LCIA spatially-explicit characterization factors as those become more common. Users and LCA practitioners are encouraged to suggest additional LCIA methods or applications of the lciafmt as described in the package Wiki.

## Figures and Tables

**Table 1:** Impact methods processed by lciafmt (Young et al., 2021d, 2021c, 2021b, 2021a).

Method	Indicators	Characterization Factors (Source)	Characterization Factors (Mapped to FEDEFL)
TRACI2.1	9 midpoint	33,429	200,686
ReCiPe2016	18 midpoint and 22 endpoint across three perspectives	347,010	2,429,470
ImpactWorld+	18 midpoint and 26 endpoint	87,804	457,983
FEDEFL Inventory Methods	9 indicators	n.a.	5,649
